# Exclusive expression of KANK4 promotes myofibroblast mobility in keloid tissues

**DOI:** 10.1038/s41598-024-59293-z

**Published:** 2024-04-16

**Authors:** Mayumi Oishi, Keiko Shinjo, Keisuke Takanari, Ayako Muraoka, Miho M. Suzuki, Miki Kanbe, Shinichi Higuchi, Katsumi Ebisawa, Kazunobu Hashikawa, Yuzuru Kamei, Yutaka Kondo

**Affiliations:** 1 Department of Plastic and Reconstructive Surgery, Aichi Children’s Health and Medical Center, Obu, Japan; 2 Department of Plastic and Reconstructive Surgery, Nagoya University Graduate School of Medicine, 65 Tsurumai-Cho, Showa-Ku, Nagoya, 466-8560 Japan; 3 Division of Cancer Biology, Nagoya University Graduate School of Medicine, 65 Tsurumai-Cho, Showa-Ku, Nagoya, 466-8550 Japan; 4 Division of Plastic and Reconstructive Surgery, Aichi Cancer Center Research Institute, Nagoya, Japan; 5 Department of Obstetrics and Gynecology, Nagoya University Graduate School of Medicine, Nagoya, Japan; 6 Center for One Medicine Innovative Translational Research (COMIT), Nagoya University, Nagoya, Japan; 7 Institute for Glyco-Core Research (iGCORE), Nagoya University, Nagoya, Japan

**Keywords:** Diseases, Skin diseases

## Abstract

Keloids are characterized by abnormal wound healing with excessive accumulation of extracellular matrix. Myofibroblasts are the primary contributor to extracellular matrix secretion, playing an essential role in the wound healing process. However, the differences between myofibroblasts involved in keloid formation and normal wound healing remain unclear. To identify the specific characteristics of keloid myofibroblasts, we initially assessed the expression levels of well-established myofibroblast markers, α-smooth muscle actin (α-SMA) and transgelin (TAGLN), in scar and keloid tissues (n = 63 and 51, respectively). Although myofibroblasts were present in significant quantities in keloids and immature scars, they were absent in mature scars. Next, we conducted RNA sequencing using myofibroblast-rich areas from keloids and immature scars to investigate the difference in RNA expression profiles among myofibroblasts. Among significantly upregulated 112 genes, KN motif and ankyrin repeat domains 4 (*KANK4*) was identified as a specifically upregulated gene in keloids. Immunohistochemical analysis showed that KANK4 protein was expressed in myofibroblasts in keloid tissues; however, it was not expressed in any myofibroblasts in immature scar tissues. Overexpression of KANK4 enhanced cell mobility in keloid myofibroblasts. Our results suggest that the KANK4-mediated increase in myofibroblast mobility contributes to keloid pathogenesis.

## Introduction

The term keloid refers to a group of fibroproliferative skin diseases characterized by excessive accumulation of myofibroblasts and extracellular matrix (ECM)^1^. Keloids grow beyond the boundaries of the original wound site and invade the surrounding tissue^2^. They develop after different skin damage (e.g., scratches, insect bites, vaccination, surgical wounds) and significantly affect the quality of life of individuals^3^. The involvement of several factors, such as genetic predisposition (e.g., a high prevalence in African and Asian ancestry^4^), hormonal influences, and wound tension has been proposed. Nevertheless, the pathogenesis of keloids is not fully understood^5^.

In the normal wound healing process, fibroblasts are activated and differentiate into myofibroblasts through the stimulation of transforming growth factor-β (TGF-β)^6^. Subsequently, myofibroblasts secrete a large amount of ECM to produce contractile new connective tissue termed granulation tissue. In the final step, the number of myofibroblasts is reduced by apoptosis, and the connective tissue is remodeled into the structure of mature scars^7,8^. Persistent myofibroblasts activation and excessive ECM accumulation contribute to keloid formation^4^. Recent single-cell RNA sequencing (scRNA-seq) studies identified four different subclasses of fibroblasts subclasses in keloids^9^. Among them, the number of mesenchymal fibroblasts (a type of myofibroblasts) characterized by collagen type XI alpha 1 chain (*COL11A1*) and periostin (*POSTN*) expression, was increased in keloid compared with normal scars. *POSTN* is an important gene involved in keloid formation and is associated with collagen expression. However, expression of *COL11A1* and *POSTN* is also observed during the normal wound healing process^10–12^ and is not specific to keloid formation. Thus, the processes of keloid formation and the normal wound healing are very similar. However, uncertainty persists regarding whether the myofibroblasts implicated in keloid formation differ from those involved in normal wound healing in terms of cell populations and gene expression patterns, or if they represent the same type of fibroblasts.

In this study, we investigated differences in the features of myofibroblasts in different scars. For this purpose, we used myofibroblast-rich regions from immature scars and keloids, and performed RNA-seq. The results suggested the presence of distinct characteristics between myofibroblasts in keloids and those in immature scars, with *KANK4* emerging as a specifically upregulated gene in keloids. Immunohistochemical analysis confirmed the expression of KANK4 protein in myofibroblasts within keloid tissues, whereas it was absent in myofibroblasts in immature scar tissues.

*KANK* genes are characterized by ankyrin-repeat and coiled-coiled domains, and a N-terminal KN motif. This family genes consists of four members (i.e., KANK1-4). It has been reported that these proteins are involved in cell migration and adhesion^13–15^. *KANK1* was initially identified as a candidate tumor suppressor gene in renal cell carcinoma^16^. In human embryonic kidney HEK-293 cells and mouse embryonic fibroblasts, Kank1 binds to 14-3-3 proteins. This binding is enhanced by the phosphorylation of Kank1 by Akt. Kank1 inhibits RhoA activation by binding to 14-3-3 proteins and negatively regulates the formation of actin stress fibers, thereby leading to the inhibition of cell migration^17^. Studies in kidney podocytes showed that KANK2 repressed RhoA activity. However, KANK4 did not exert an effect on the active state of RhoA and Rac family small GTPase 1 (RAC1)^18^. These data suggested that each KANK protein has a different impact on cell movement, which might depend on the cell type and the location of the cell^19^.

Furthermore, through further analysis showed that overexpression of KANK4 enhance cell mobility in keloid myofibroblasts. These findings suggest that the KANK4-mediated increases in myofibroblast mobility contribute to keloid pathogenesis. Our discoveries may provide potential therapeutic targets for keloids in the future.

## Materials and methods

### Human tissue samples

Samples from keloid, normal scars, and normal skin sample were collected from patients who underwent surgery at the Department of Plastic and Reconstructive Surgery of Nagoya University Hospital (Nagoya, Japan) from 2003 to 2022. The keloid samples used in this study were collected from 57 patients. All patients showed evidence indicating the presence of keloids, including clinical appearance, history, and pathological examination. The normal scar samples (n = 82) were collected from patients without symptoms or clinical appearance of keloids. We further classified normal scar samples into two subclasses: immature scars, i.e., scars that formed within 3 years after injury (n = 72); and mature scars, i.e., scars that formed more than 3 years after injury (n = 10). A single normal skin sample was collected to establish a normal fibroblast cell line from residual donor tissue following reconstructive surgery. Detailed patient information is provided in Table 1 and Supplementary Table 1. The majority of mature and immature scars were obtained from planned surgeries associated with breast reconstructions. Therefore, most scar samples were obtained from the chest of females. The collection of samples for this study was approved by the ethics review committee of Nagoya University Hospital (approval number: 2019-0079-5). Written informed consent was provided by the participants in accordance with relevant guidelines and regulations. Samples were collected from June 10, 2019, to December 28, 2022. Archived samples were used for Immunohistochemical analysis, and the clinical records were reviewed on July 1, 2019.

**Table 1:** Clinical background of keloid patients and normal scar controls.

	Normal scars		Keloids
	Mature scars	Immature scars	
	n = 10	n = 72	n = 57
Age (years)	41.6 ± 17.15	50.2 ± 13.5	39.9 ± 19.9
Male/female ratio	2:8	8:64	21:36
Location			
Ear	0	0	24
Chest	6	57	4
Abdomen	1	5	19
Others	3	10	10
Duration after injury (months)	90.6 ± 56.3	13.7 ± 6.7	68.8 ± 74.2

Ages and durations after injury are presented as the mean ± standard deviation (SD).

### Immunohistochemistry

Formalin-fixed, paraffine-embedded tissue sections were deparaffinized and subjected to antigen retrieval using tris/EDTA buffer, pH9.0 (ImmunoActive IA9500; Matsunami Glass, Osaka, Japan). After blocking in 3% H_2_O_2_ for 20 min, specimens were incubated with the primary antibodies for overnight: transgelin (TAGLN; ab14106, 1:200; abcam, Cambridge, UK), α-smooth muscle actin (α-SMA; M0858, 1:100; Dako, Glostrup, Denmark), KN motif and ankyrin repeat domains 4 (KANK4; ab121410, 1:50; abcam), opioid binding protein/cell adhesion molecule like (OPCML; ab238143, 1:1,000; abcam), S100 calcium binding protein A7 (S100A7; #45298, 1:400; Cell Signaling Technology, Danvers, MA, USA). Subsequently, the specimens were incubated with horseradish peroxidase-conjugated (HRP-conjugated) secondary antibody and subjected to 3,3′-diaminobenzidine (Nichirei Bioscience, Tokyo, Japan) and hematoxylin staining. Frozen tissue samples were also used for TAGLN staining. Binarized images of KANK4 staining were processed and quantified by the ImageJ software (https://imagej.net/ij/).

### RNA extraction

Total RNA from the cell lines was extracted using TRIzol (Thermo Fisher Scientific, Waltham, MA, USA). To obtain RNA from clinical tissue samples, frozen sections were stained with anti-TAGLN antibody to visualize TAGLN-positive cells in tissue samples. Subsequently, areas of interest containing TAGLN-positive fibroblasts were manually dissected from unstained serial sections using microtweezers under a magnifier at – 30 °C. The total RNA from the obtained tissues was extracted using ReliaPrep RNA Tissue Miniprep System (Promega, Madison, WI, USA). Briefly, tissues were homogenized using a hand homogenizer in TRIzol reagent (Thermo Fisher Scientific) and centrifuged. The supernatant was mixed with isopropanol and passed through a column provided with the kit. After several wash steps and DNase treatment, total RNA was eluted in RNase-free water. All RNA samples were evaluated for quality and quantified using a Nanodrop 2000 spectrophotometer (Thermo Fisher Scientific). RNA samples were stored at – 20 °C.

### RNA-seq

Libraries were constructed using the QIAseq UPX 3′ Transcriptome Kit (QIAGEN, Hilden, Germany) according to the instructions provided by the manufacturer. The samples were analyzed using a NovaSeq 6000 instrument (Illumina, Santa Clara, CA, USA). RNA-seq Analysis Portal 2.0 (QIAGEN) was used to map the sequencing reads to a human reference genome (GRCh38). Data analysis was performed on the RNA-seq analysis portal (QIAGEN). Details of the analysis workflow, experiment summary and quality control are available in the Supplementary Table 4. The raw FASTQ files were deposited in the Gene Expression Omnibus database under accession number GSE245660. mRNA expression data of the GSE113619 and GSE92566 datasets were analyzed. Statistical significance for differential gene expression was determined using a fold change threshold of > 2 and a false discovery rate P-value of ≤ 0.1 when comparing the current study and GSE113619, and a fold change threshold of > 2 and a P-value of ≤ 0.05 when comparing GSE113619 and GSE92566. scRNA-seq data of the GSE163973 dataset were obtained from Gene Expression Omnibus database. Sequence data was reanalyzed in the same manner as the original study^9^. Briefly, sequencing reads were quality assessed and transcripts were mapped to a reference human genome (hg38) and then assigned to individual cells using the Cell Ranger pipeline (10 × Genomics). From the UMI counting matrices, gene expression levels were normalized and principal component analysis (PCA) was performed. Clustering was then performed on the PCA scores using significant PCs, and cell subpopulations were identified using the Louvain method. Visualization was performed using Uniform Manifold Approximation and Projection (UMAP), and fibroblast subpopulations were identified using cluster-specific marker genes, COL1A1 and COL3A1.

### Quantitative reverse transcription polymerase chain reaction (reverse transcription-PCR)

RNA was reverse-transcribed with the Prime Script RT Master Mix (Takara Bio, Shiga, Japan) according to the instructions provided by the manufacturer. TaqMan PCR and SYBR Green quantitative PCR were carried out in triplicate for the target genes. The expression levels of target genes were determined using the delta-delta Ct (cycle threshold) method and normalized to those of glyceraldehyde-3-phosphate dehydrogenase (GAPDH). Oligonucleotide primers of GAPDH for TaqMan PCR assays (Hs.PT.39a.22214836; Integrated DNA Technologies, Coralville, IA, USA) and the primer sets for SYBR Green assays are shown in Supplementary Table 2.

### Cell culture

Primary fibroblasts were established as previously described^20^. Samples were collected from two scar tissues (i.e., one from a keloid and another from an immature scar). The dermis was isolated from the epidermis, sectioned into fragments (dimensions: approximately 2 mm × 2 mm) using surgical blades, and digested with 100 units/ml collagenase type I (Gibco, Carlsbad, CA, USA) at 37 °C for 24 h. The mixture was centrifuged at 1200 rpm for 3 min, the supernatant was discarded, and cell pellets were harvested. Cells were cultured in Dulbecco’s modified Eagle’s medium (DMEM; Wako, Osaka, Japan) containing 10% fetal bovine serum (FBS; Thermo Fisher Scientific) and 1% penicillin–streptomycin (Wako) at 37 °C in a humidified incubator with 5% CO_2_. The medium was changed every 3 days; when the cells reached 90% confluence, they were split at a 1:2 ratio. The established cell lines were cultured for a maximum of 10 passages. SC10 was established in a previous study^21^ and maintained in DMEM/F12 (Wako) supplemented with 10% FBS. TGF-β (#100-21, PeproTech, Cranbury, NJ, USA) was used at a concentration of 10 ng/µl.

### Construction of the KANK4 expression vector

A cDNA fragment of human KANK4 (NM_001320269.2) was amplified by PCR using cDNA of primary cultured fibroblasts obtained from a patient with keloid. KOD-Plus-Neo (TOYOBO, Osaka, Japan) was used in this experiment, along with the following primers: 5′-CGAGCATGCATCTAGTATGGAGAAGACAGATGAGAT-3′ and 5′-AATAGGGCCCTCTAGCTACAGCCCCAGGGACCT-3′. The DNA fragment was subsequently inserted into a pcDNA3 vector (Thermo Fisher Scientific) containing enhanced green fluorescent protein (EGFP) to construct pcDNA3-EGFP-KANK4. The *KANK4* sequence in the construct was verified by Sanger sequencing. The pcDNA3-EGFP vector was used as a control. The human KANK4 (NM_181712.5) expression vector was kindly provided by Dr. Kiyama (Kyushu Sangyo University, Japan)^13,14^.

### Cell manipulation

Primary fibroblasts were seeded in six-well plates at a density of 2 × 10^5^ cells/well for 24 h before transfection using the KANK4 vector. Cells were transfected with 2.5 µg of pcDNA3-EGFP or pcDNA3-EGFP-KANK4 using Lipofectamine 3000 (Thermo Fisher Scientific) for 48 h. Thereafter, cells were plated for migration assay or proliferation assays. After transfection, SC10 cells were trypsinized and resuspended in phosphate-buffered saline, and subjected to fluorescence activated cell sorting (FACS) analysis with FACSMelody (Becton & Dickinson, Franklin Lakes, NJ, USA). GFP-positive cells were collected and cultured for 7 days to be used in further experiments. To knockdown KANK4, we treated the cell with 100 nmol/L of siKANK4 or negative control siRNA (Silencer Select Negative Control #1 siRNA,4390844, Thermo Fisher Scientific) using Lipofectamine 3000 (Thermo Fisher Scientific) for 48 h. siKANK4 sequence; sense, UUUUCAUUAUGGAUUUAAGGC and antisense, CUUAAAUCCAUAAUGAAAAAG.

### Western blot analysis

Cell lysates were extracted from fibroblasts following transfection with the pcDNA3-EGFP vector or pcDNA3-EGFP-KANK4 vector. Total protein samples (50 µg) were run on 7.5% sodium dodecyl sulfate–polyacrylamide gel electrophoresis gels and transferred onto nitrocellulose membranes. Subsequently, the membrane were incubated with the following antibodies primary antibodies: rabbit polyclonal anti-KANK4 (ab121410; abcam), mouse monoclonal anti-GFP (M048-3; MBL, Tokyo, Japan), and mouse monoclonal anti-β-actin (#3700; Cell Signaling Technology). Thereafter, the membranes were incubated with secondary antibodies HRP-linked anti-mouse immunoglobulin G (IgG) (7076S; Cell Signaling Technology) and HRP-linked anti-rabbit IgG (7074S; Cell Signaling Technology). The ImageQuant LAS 500 (Cytiva, Marlborough, MA, USA) was used to detect proteins visualized using ECL Prime Western Blotting Detection Reagent (Cytiva).

### Transwell migration assay

Migration assays were performed using chambers with membranes containing 8-μm pore Cell Culture Inserts (CORNING, Corning, NY, USA). The lower chambers were filled with DMEM containing 20% FBS. At 48 h after vector transfection, 1 × 10^5^ cells were seeded into the upper chamber containing medium without FBS. After 24 h of incubation, cells that had migrated and attached to the bottom membrane were stained with 0.1% crystal violet. Stained cells were counted in four randomly selected fields in each well using the ImageJ software.

### Cell proliferation assay

Cells were seeded in 96 well plates at a density of 5 × 10^4^ cell/well. Cell growth was assessed using Cell Count Reagent SF (Nacalai Tesque, Kyoto, Japan).

### Statistical analysis

Data are presented as the mean ± standard deviation. *P*-values < 0.05 indicate statistically significant differences. Differences between two groups were compared using the unpaired *t*-test. One-way analysis of variance was used for multiple comparisons. All reported *P*-values were two-tailed. Statistical analyses were performed using the GraphPad Prism version 9 software (GraphPad Software, San Diego, CA, USA).

## Results

### Myofibroblasts characteristics varied between normal scars and keloids

**Figure 1:**
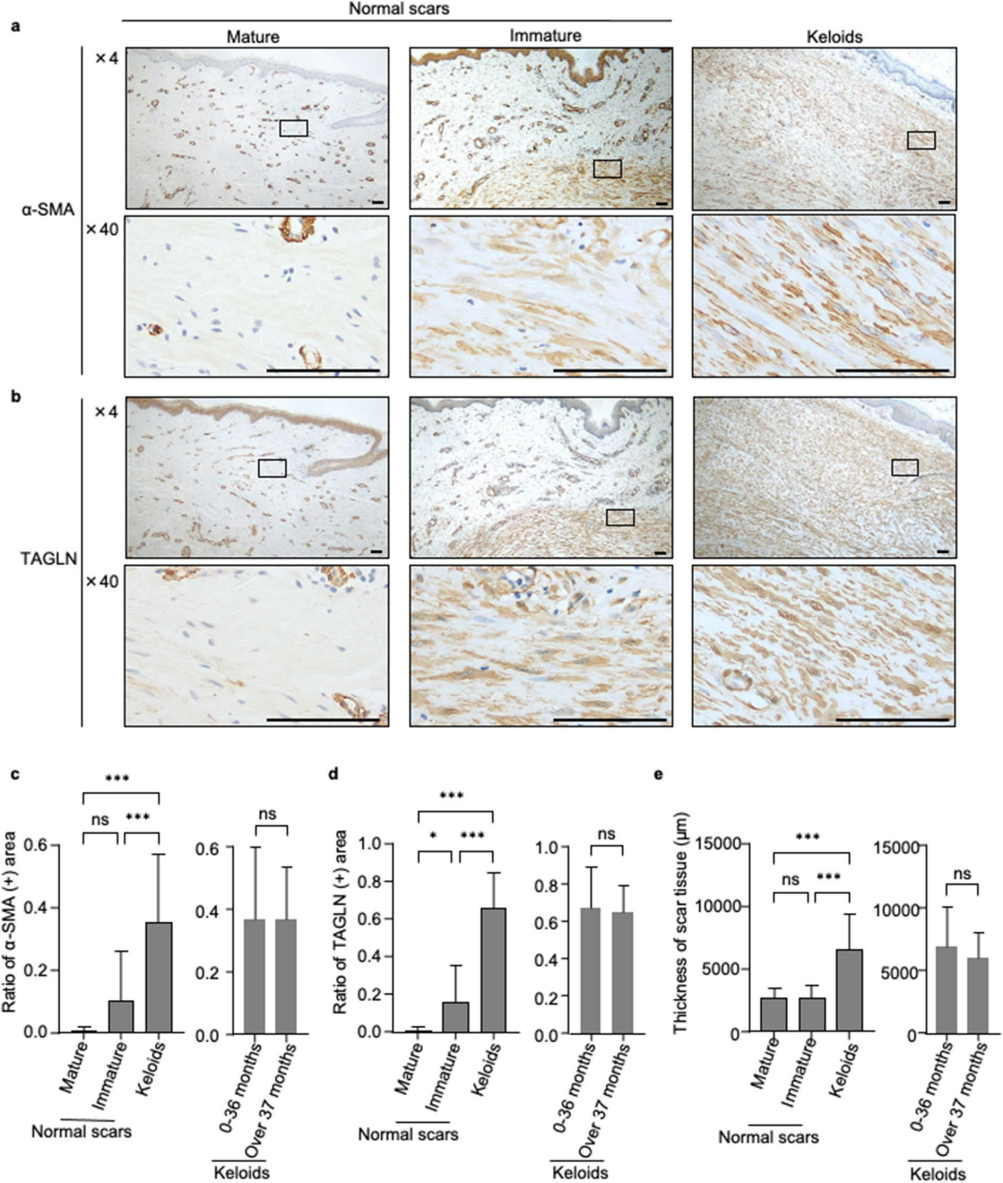
Distribution of myofibroblasts of normal scars and keloids. (**a**,**b**) Representative image of immunohistochemical analyses for α-SMA (**a**) and TAGLN (**b**) in mature scars, immature scars and keloids. Rectangle areas are magnified in the second row (magnification 40×). Scale bar: 100 μm. (**c**) Left panel: Percentage of areas with α-SMA-positive myofibroblasts in the whole scars. Scars were grouped as mature scars (n = 10), immature scars (n = 51), and keloids (n = 44). Right panel: Keloid samples were further divided into two subgroups, namely those formed within 36 months after injury (n = 24) and those formed after 37 months (n = 16). Error bars show the mean ± SD. *P*-value was determined suing the two-tailed *t*-test. ***P < 0.001. (**d**) Left panel: Percentage of areas with TAGLN-positive myofibroblasts in the whole scars. Scars were grouped as mature scars (n = 10), immature scars (n = 53), and keloids (n = 51). Right panel: Keloid samples were further divided in to two subgroups, namely those formed within 36 months after injury (n = 27) and those formed after 37 months (n = 19). Error bars show the mean ± SD. *P*-value was determined using the two-tailed *t*-test. *P < 0.05, ***P < 0.001. (**e**) Left panel: Thickness of scar tissue. Distances was determined by measuring the perpendicular distance from the bottom of the epidermal basal layer to the adipose layer. Five different fields were used for measurement in each sample, and average distance was used for calculation. Scars were grouped as mature scars (n = 10), immature scars (n = 53) and keloids (n = 51; left). Right panel: Keloid samples were further divided into two subgroups, namely those formed within 36 months after injury (n = 27) and those formed after 37 months (n = 19). Error bars show the mean ± SD. *P*-value was determined using the two-tailed *t*-test. ***P < 0.001. *α-SMA* α-smooth muscle actin, *ns* not significant, *SD* standard deviation, *TAGLN* transgelin.

Initially, we conducted immunohistochemical analysis focusing on representative myofibroblast markers α-SMA^22^ and TAGLN^23^ to examine the distribution of myofibroblasts within normal scar and keloid samples (n = 63 and 51, respectively). Notably, α-SMA- and TAGLN-positive myofibroblasts were abundantly present in both normal immature scar and keloid samples. In contrast, such myofibroblasts were not found in mature scar samples (Fig. 1a,b). Furthermore, we calculated the ratio of the myofibroblast-positive area to the total scar area (Supplementary Fig. 1). The areas of α-SMA-positive or TAGLN-positive myofibroblasts in keloid tissues were larger than those observed in mature and immature scar tissues (*P* < 0.001) (Fig. 1c,d, left panel). Comparison of mature and immature scar tissues revealed more significant difference in the area of TAGLN-positive myofibroblasts than in that of α-SMA-positive myofibroblasts (*P* < 0.05 and *P* = 0.2916, respectively). In keloid samples, the duration of disease (< 3 years vs. > 3 years) did not influence the expression of α-SMA and TAGLN (Fig. 1c,d, right panel).

Next, we quantified the thickness of the scar tissue. Keloid samples showed a considerably thicker disease area compared with immature or mature scars (*P* < 0.001) (Fig. 1e, left panel). Among keloid samples, there was no difference in the thickness of the disease area based on the duration of disease.

These results suggested that the number of α-SMA- and TAGLN-positive myofibroblasts in normal scars diminishes over time after injury. In contrast, the widely distributed α-SMA and TAGLN-positive myofibroblasts compose a thick keloid lesion. The thickness of the disease area of keloids was not affected by the duration of disease.

### RNA-seq reveals the distinct characteristics of keloid myofibroblasts

**Figure 2:**
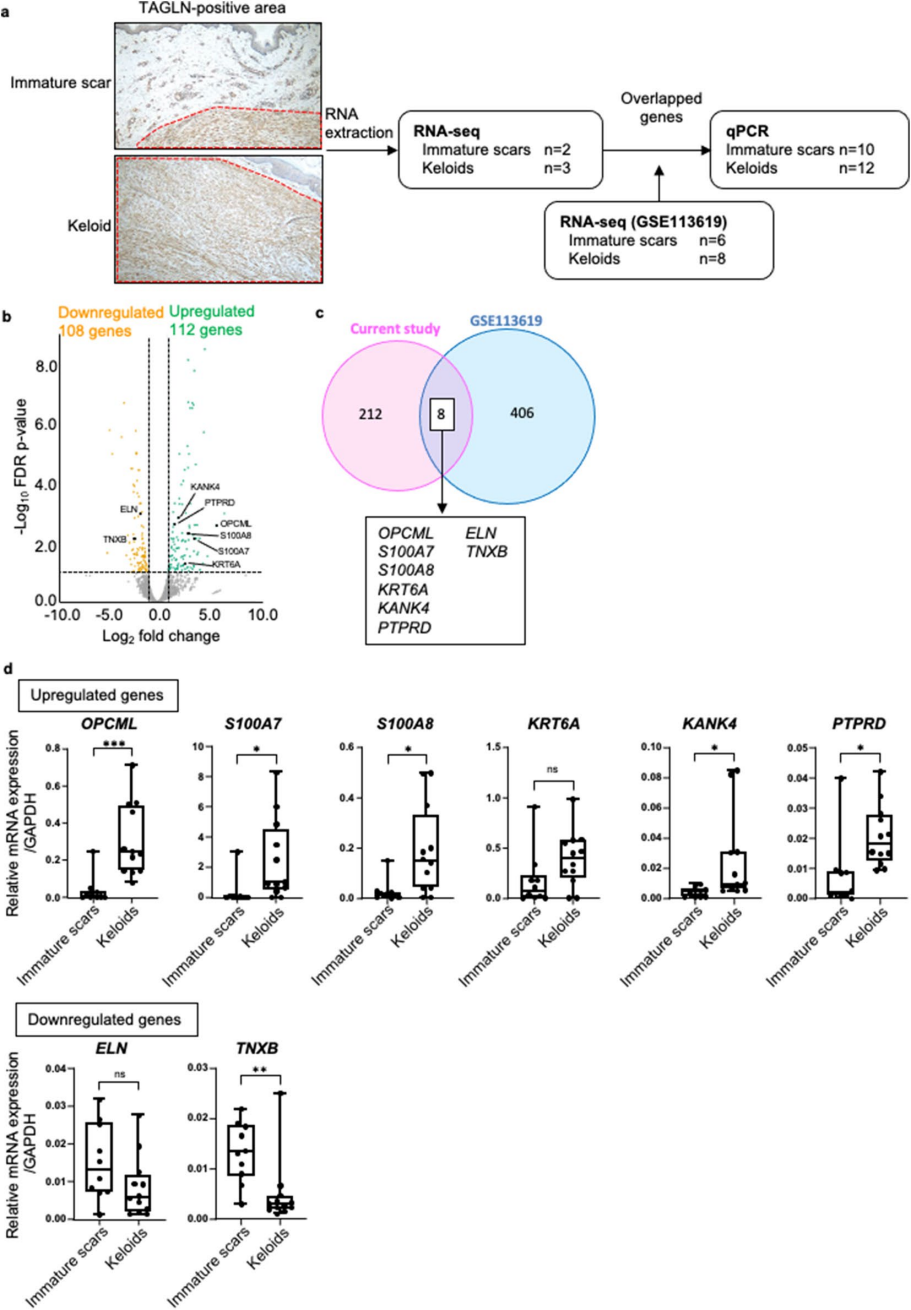
3′ RNA-seq analysis identified keloid specific differentially expressed genes. (**a**) Scheme of dissections of the TAGLN-positive area, and workflow of the analysis performed in this study. (**b**) 3′ RNA-seq analysis for immature scars (n = 2) and keloids (n = 3). Volcano plots showing the differentially 112 upregulated genes and 108 downregulated genes in keloids (fold change > 2, FDR *P*-value ≤ 0.1). (**c**) Venn diagram showing the eight shared differentially expressed genes among the aforementioned 220 genes identified though the current 3′ RNA-seq analysis and 414 genes detected in the GSE113619 dataset (fold change > 2, FDR *P*-value ≤ 0.1). (**d**) Relative mRNA expressions of the eight differentially expressed genes in immature scars (n = 10) and keloids (n = 12) compared with GAPDH. Error bars show the mean ± SD. *P*-value was determined using the two-tailed *t*-test. *P < 0.05, **P < 0.01, ***P < 0.001. *ELN* elastin, *FDR* false discovery rate, *GAPDH* glyceraldehyde-3-phosphate dehydrogenase, *KANK4* KN motif and ankyrin repeat domains 4, *KRT6A* keratin 6A, *OPCML* opioid binding protein/cell adhesion molecule like, *PTPRD* protein tyrosine phosphatase receptor type D, *qPCR* quantitative polymerase chain reaction, *RNA-seq* RNA sequencing, *S100A7* S100 calcium binding protein A7, *TNXB* tenascin XB, *S100A8* S100 calcium binding protein A8, *SD* standard deviation, *TAGLN* transgelin.

Next, we analyzed differences in the transcriptome of myofibroblasts between keloids and immature scars. It has been reported that TAGLN is a better marker of smooth muscle differentiation than α-SMA in myofibroblasts^24,25^. Therefore, we collected the TAGLN-positive area from three keloid samples and two immature scar samples, and performed RNA-seq (Fig. 2a, Supplementary Fig. 2, Supplementary Table S1). We found that 112 genes were significantly upregulated and 108 genes were downregulated in keloid samples compared with immature scar samples (fold change > 2, false discovery rate *P*-value ≤ 0.1) (Fig. 2b). Furthermore, we analyzed the public RNA-seq dataset of keloids samples obtained from keloid-prone patients (n = 8) and immature scar samples from healthy individuals (scars 42 days after injury, n = 6) (GSE113619)^26^. This analysis revealed 192 upregulated genes and 222 downregulated genes in keloids compared with immature scars. Furthermore, we compared the results obtained from the analysis of these two datasets and identified an overlap of six upregulated and two downregulated genes (Fig. 2c, Table 2).

**Table 2:** Differentially expressed genes from RNA-seq analysis.

Gene name	Ensembl ID	Keloids vs immature scars in TAGLN-positive area		Keloids vs immature scars (GSE113619)	
		Log_2_ FC	FDR *P*-value	Log_2_ FC	FDR *P*-value
*OPCML*	ENSG00000183715	6.068008	0.002773	1.646878	0.000008
*S100A7*	ENSG00000143556	3.799032	0.007586	1.848036	0.003107
*S100A8*	ENSG00000143546	3.238459	0.005247	1.872452	0.003553
*KRT6A*	ENSG00000205420	2.700261	0.056262	1.209893	0.039475
*KANK4*	ENSG00000132854	2.083482	0.001527	1.047065	0.015044
*PTPRD*	ENSG00000153707	1.663727	0.002446	1.030291	0.003408
*ELN*	ENSG00000049540	− 1.760537	0.001097	− 1.427264	0.000002
*TNXB*	ENSG00000168477	− 2.356346	0.007458	− 1.139182	0.000113

*FC* fold change, *FDR* false discovery rate.

We also obtained data from another dataset (GSE92566), which included four keloid samples and three normal skin samples derived from keloid-prone patients. A microarray platform was utilized for this analysis. Among the 770 significantly upregulated genes and 1120 downregulated genes in keloids from GSE92566, 18 upregulated genes and 11 downregulated genes overlapped with those detected in GSE113619 (fold change > 2, *P*-value ≤ 0.05) (Supplementary Table S3). Analysis of these three datasets (i.e., current RNA-seq, GSE113619, and GSE92566) showed recurrent upregulation of *OPCML, S100A7, KANK*), and protein tyrosine phosphatase receptor type D *(PTPRD*).

Subsequently, we then verified the expression levels of these eight overlapped genes (Fig. 2c, Table 2) in RNA extracted from the TAGLN-positive area of keloid (n = 12) and immature scar (n = 10) samples by quantitative PCR (Fig. 2d, Supplementary Table S1). Of those, six genes (i.e., *OPCML, S100A7,* S100 calcium binding protein A8 [*S100A8*]*, KANK4, PTPRD,* tenascin XB [*TNXB*]) were significantly differentially expressed between keloid and immature scar samples (Fig. 2d).

### KANK4 protein was specifically expressed in keloid myofibroblasts

We sought to verify whether the proteins of the six genes that characterize the TAGLN-positive areas of keloids (Fig. 2d) were expressed by fibroblasts in the tissue of keloids and immature scars. *OPCML* exhibited the greatest upregulation among those genes in keloids (Table 2). We performed immunohistochemical analysis of OPCML in keloids and immature scars, and found that fibroblasts in both tissues were not stained with OPCML (Supplementary Fig. 3a). In keloids, only sebaceous glands were slightly stained with OPCML.

*S100A7* and *S100A8* exhibited the second and third greatest upregulation in keloids, respectively. S100 refers to a family of proteins expressed in different tissues and cells. According to the scRNA-seq data in the Human Protein Atlas (https://www.proteinatlas.org/), *S100A8* is mostly expressed in keratinocytes and macrophages in skin, while *S100A7* is expressed in fibroblasts and keratinocytes. Therefore, we conducted an immunohistochemical analysis of S100A7 using the same set of samples. We found that cutaneous appendages, such as sebaceous glands and hair follicles in keloid tissues, were stained with S100A7. However, fibroblasts were not stained in both keloids and immature scars (Supplementary Fig. 3b).

**Figure 3:**
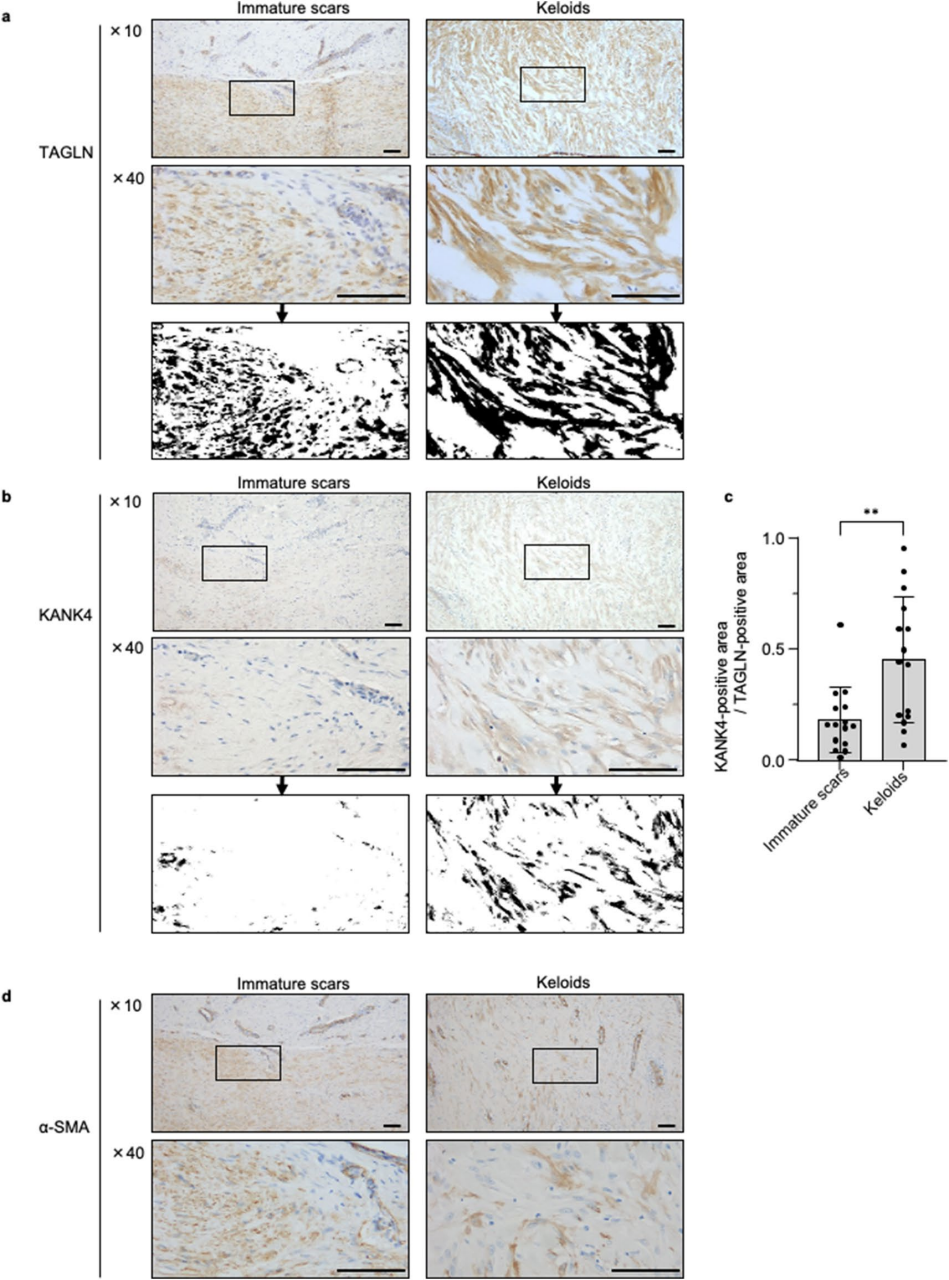
KANK4 protein was specifically expressed in keloid myofibroblasts. (**a**,**b**) Representative image immunohistochemical analyses for TAGLN (**a**) and KANK4 (**b**) using serial sections of the TAGLN-positive areas of normal scars and keloids. Magnification: × 10 and × 40. Scale bar: 100 μm. The bottom images show the binarized images from × 40 magnified images. (**c**) Ratio of the KANK4-positive area to the TAGLN-positive area, calculated using binarized imaged in immature scars (n = 15) and keloids (n = 15). Error bars show the mean ± SD. *P*-value was determined using the two-tailed *t*-test. **P < 0.01. (**d**) Representative image immunohistochemical analyses for α-SMA. Magnification: × 10 and × 40. Scale bar: 100 μm. *KANK4* KN motif and ankyrin repeat domains 4, *TAGLN* transgelin, *SD* standard deviation.

Next, we focused on *KANK4* (i.e., one of the upregulated genes in keloids). Immunohistochemical analysis showed that KANK4 protein was mostly expressed in TAGLN-positive myofibroblasts in keloid tissues (Fig. 3a,b). Interestingly, TAGLN-negative fibroblasts did not express KANK4 in keloid samples (Supplementary Fig. 3c). Contrarily, in immature scars, KANK4 was not expressed in any myofibroblasts. To the quantify the KANK4-positive area, we initially binarized the stained areas of TAGLN and KANK4 in consecutive sections into black and white with a threshold. Subsequently, we calculated the stained area (Fig. 3a,b). Our findings revealed a significantly higher ratio of KANK-positive area to the TAGLN-positive area in keloids samples compared with immature scar samples (*P* < 0.01) (Fig. 3c). Most of the α-SMA-positive fibroblasts also exhibited positive expression of KANK4 (Fig. 3d).

Indeed, scRNA-seq data of keloid and normal scar fibroblasts (GSE163973) showed the presence of TAGLN-positive myofibroblasts in both types of fibroblasts. Of note, KANK4- positive fibroblasts are distinct from normal scar fibroblasts (Supplementary Fig. 3d,e).

These data indicate that myofibroblasts in keloids represent distinct cell populations compared with those found in immature scars, as inferred from the pattern of KANK4 expression.

### Upregulation of KANK4 promoted cell mobility in skin fibroblasts

**Figure 4:**
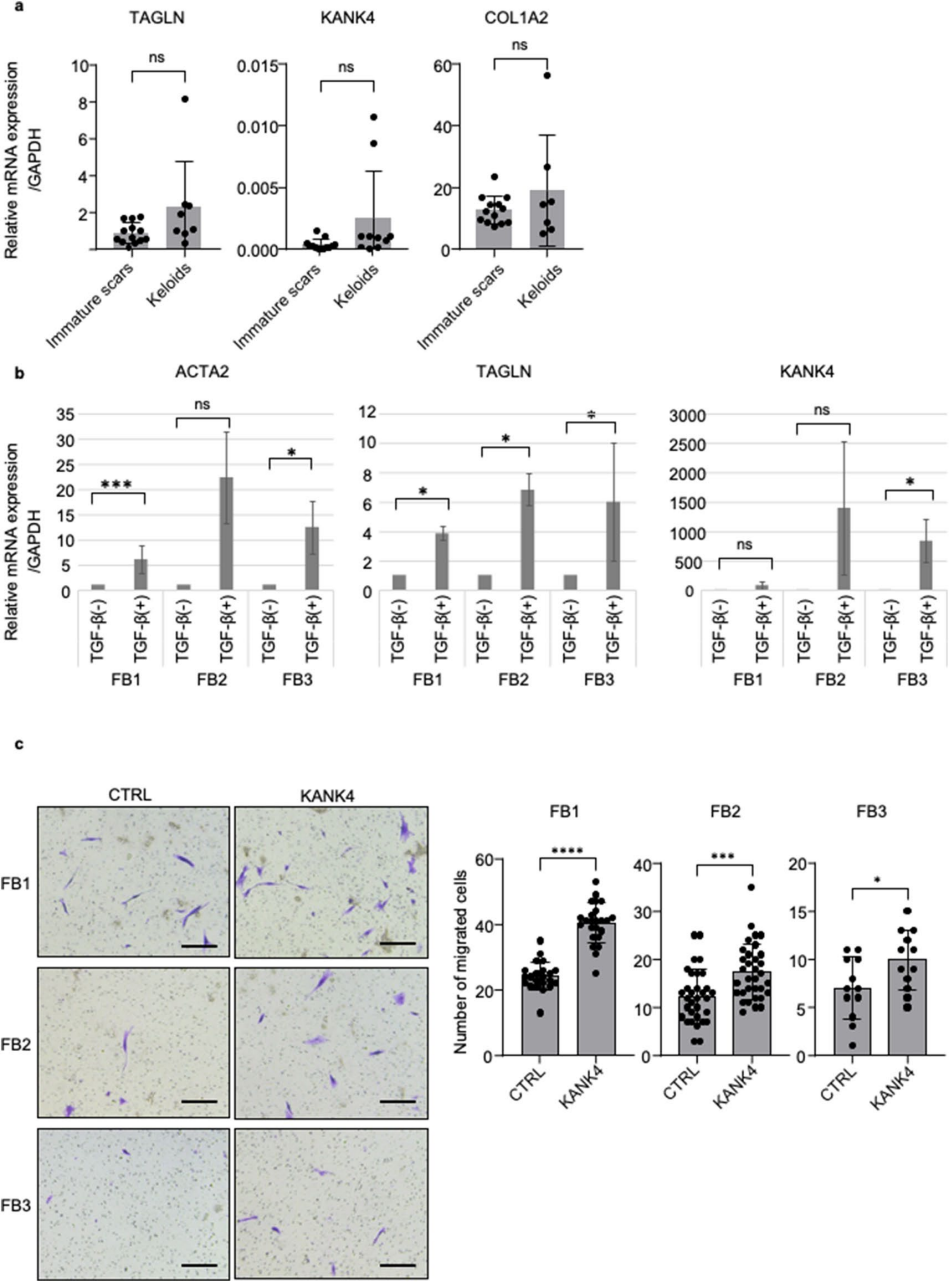
KANK4 promoted cell mobility of fibroblasts. (**a**) mRNA expression levels of established primary fibroblasts from immature scar (n = 13) and keloid (n = 10) tissues. Error bars show the mean ± SD. *P*-value was determined using the two-tailed *t*-test. (**b**) mRNA expression levels of *ACTA2*, *TAGLN*, and *KANK4* after treatment with TGF-β (10 ng/μl) for 24 h. Error bars show the mean ± SD. *P*-value was determined using the two-tailed *t*-test. *P < 0.05. (**c**) Representative image of migrated cells in Transwell assay after transfection of cells with pcDNA3-EGFP (CTRL) or pcDNA3-EGFP-KANK4 (KANK4) for 48 h. Scale bar: 250 μm (left). The bar graphs show the number of migrated cells, counted in each field under a microscope (right). Error bars show the mean ± SD. *P*-value was determined using the two-tailed *t*-test. ***P < 0.001, ****P < 0.0001. *ACTA2* actin alpha 2, *COL1A2* collagen type I alpha 2 chain, *CTRL* control, *EGFP* enhanced green fluorescent protein, *KANK4* KN motif and ankyrin repeat domains 4, *ns* not significant, *TAGLN* transgelin, *TGF-β* transforming growth factor-β, *SD* standard deviation.

We established fibroblasts from immature scar and keloid tissues to investigate the function of KANK4 in these cells. The expression levels of *TAGLN* and *KANK4* tend to be higher in keloid fibroblasts (n = 10) versus immature scar fibroblasts (n = 13) (*P* = 0.06 and 0.06, respectively) (Fig. 4a). Interestingly, the expression of collagen type I alpha 2 chain (*COL1A2*) was not significantly different between keloid fibroblasts and immature scar fibroblasts (*P* = 0.23) (Fig. 4a). Actin alpha 2 (*ACTA2*, gene which code α-SMA) and *TAGLN* are well-established genes; their expression is upregulated by TGF-β in fibroblasts. Therefore, we treated fibroblasts established from normal skin (NFB1), immature scar (FB1) and keloid (FB2 and FB3) tissue with TGF-β, and assessed the mRNA expression levels of these genes. *ACTA2* and *TAGLN* expression was upregulated in all types of fibroblasts after stimulation with TGF-β. *KNAK4* expression was also upregulated by TGF-β in these fibroblasts; however, the increase in *KANK4* expression was markedly more significant in keloid fibroblasts (*P* = 0.30 and 0.04 in FB2 and FB3, respectively) versus immature scar fibroblasts (Fig. 4b, Supplementary Fig. 4a).

To understand the function of KANK4 in keloid fibroblasts, we overexpressed KANK4 in the established immature fibroblasts (FB1) and keloid fibroblasts (FB2, FB3) (Supplementary Fig. 4b). Since the expression level of KANK4 was very low without TGF-β stimulation (Fig. 4b), we used keloid fibroblasts for overexpression study. The results of the migration assay showed that KANK4 overexpression promoted cell mobility without affecting the rate of cell proliferation rate in both FB1, FB2 and FB3 (*P* < 0.0001, 0.0007, and 0.034, respectively) (Fig. 4c and Supplementary Fig. 4c). Next, we knockdown KANK4 with siRNA and examined the cell mobility (Supplementary Fig. 4d). Although the expression levels of KANK4 are low in stable condition, the cell mobility was significantly affected by KANK4 knockdown (Supplementary Fig. 4e). Interestingly, KANK4 overexpression did not have any impact of collagen expression (COL1A and COL3A1) (Supplementary Fig. 5a).

We utilized immortalized endometrial fibroblasts, SC10, for further study because our primary fibroblasts were unable to culture for a prolonged period after transfection with the overexpression vector^21^. SC10 are considered as myofibroblasts and express TAGLN. Following transfection with either pcDNA3-EGFP or pcDNA-EGFP-KANK4, we sorted GFP-positive cells using FACS and seeded them for migration assays (Supplementary Fig. 5b). The KANK4-overexpressing cells exhibited significantly increased mobility (*P* < 0.0001); nevertheless, there was no difference observed in cell proliferation (Supplementary Fig. 5c,d).

Thus far, two different isoforms of KANK4 have been reported, namely NM = 181,712.5 (isoform 1) and NM_001320269.2 (isoform 2). Isoform 2 lacks one exon and is shorter compared with isoform 1 (Supplementary Fig. 6a). Primer set 1 of KANK4 (amplification of the long isoform) was used in our clinical samples and cell line studies (Figs. 2d, 4a,b). Therefore, we also examined the expression of KANK4 in clinical samples using Primer set 2 (amplification of the short and long isoforms) (Supplementary Fig. 6a). The result of qPCR using Primer set 2 revealed that KANK4 expression was significantly higher in keloid tissue versus immature scar tissue (Supplementary Fig. 6b). Interestingly, the ratio of Primer set 2 to Primer set 1 was higher in keloid samples compared with immature scar samples (Supplementary Fig. 6c), suggesting that the shorter isoform of KANK4 is expressed more abundantly in keloid tissue.

Since we used the isoform 2 KANK4-overexpression vector (KANK4-vector in Supplementary Fig. 6a), we also employed the isoform 1 KANK4-overexpression vector (KANK4-full) and validated its impact on the fibroblasts^13,14^. Overexpression of the KANK4-full-length vector exerted a similar effect on cell mobility in fibroblasts to that observed following overexpression of the KANK4-vector (Supplementary Fig. 6d,e).

## Discussion

Deregulation of the normal wound healing processes following injury is considered a cause of keloid formation. Previous studies have identified numerous genes that are differentially expressed between keloids, normal skin, and normal scars. However, the key mechanisms underlying the development of keloids remain partly understood^27–29^. Recently, new technologies (e.g., scRNA-seq and spatial transcriptomics) have been used to discover novel therapeutic targets for keloids^9,30,31^. Nevertheless, due to the lack of functional studies on candidate genes, the roles of these genes in keloid formation remain unclear.

Various types of cells are involved in keloid formation; fibroblasts are primarily responsible for producing ECM and play a crucial role in the development of the keloids^4^. Therefore, in the present study, we investigated whether the phenotype of myofibroblasts differs between keloids and immature scars. We performed immunohistochemical staining using two commonly used myofibroblast markers, α-SMA and TAGLN. The results revealed the presence of α-SMA- and TAGLN-positive areas in immature scars and keloids, but not in mature scars. Additionally, the thickness of the layer which myofibroblasts were present was greater in keloids compared with immature scars. Further RNA-seq analysis, using RNA extracted from TAGLN-positive areas in keloid and immature scars, identified keloid-specific genes that showed differentially expression in these myofibroblasts. While previous scRNA-seq of keloid fibroblasts uncovered significant heterogeneity within fibroblasts populations, our analysis revealed consistently identifying the upregulation of several key genes, including *KANK4*, across not only our RNA-seq data but also two different datasets.

*KANK4* was upregulated in skin fibroblasts obtained from old donors (average age: 60 years) compared with young donors (average age: 25 years)^32^. Studies revealed an association between KANK4 and Rho GDP dissociation inhibitor α (ARHGDIA), which mediates the activation of the Rho pathway. Knockdown of *KANK4* in older fibroblasts increased RhoA activity and reduced the contractile capacity. A previous study showed that overexpression of the full-length of KANK4 reduces the formation of actin stress fibers and might have a similar function to that of KANK1, which inhibits RhoA activity^13,14^. Interestingly, we found that overexpression of both the short isoform of KANK4 and full-length KANK4 exerted similar effects on cell mobility. The function of KANK4 (i.e., inhibition or activation of cell migration) might be depend on the cell type or the cell context. In normal wound healing, KANK4-negative myofibroblasts may have a low migratory capacity. Consequently, fibroblasts remain within the original wound, contract the granulation tissue, and narrow the wound area. In keloid tissue, KANK4-positive myofibroblasts may exhibit enhanced mobility and be able to migrate beyond the original wound and expand the keloid lesion by producing ECM. Further functional studies are needed to determine the precise role of KANK4 in keloid formation.

TGF-β plays an important role in all phases of the wound healing process and is a potent activator of myofibroblasts^4^. It has been reported that keloid fibroblasts are more sensitive to TGF-β compared with normal fibroblasts^3^. Indeed, in our study, TGF-β effectively induced TAGLN and collagen expression in keloid fibroblasts. KANK4 was also induced by TGF-β in normal fibroblasts, immature scar fibroblasts and keloid fibroblasts, but the induction was much prominent in keloid fibroblast. Although, we were unable to identify the mechanism regulating KANK4 expression in keloid myofibroblasts, we hypothesized that TGF-β might be involved in this process.

The origin of KANK4-positive myofibroblasts is unclear. It is established that fibroblast precursor cells are recruited from different sources, including local resident fibroblasts, pericytes, smooth muscle cells, and fibrocytes from bone marrow^22^. KANK4-negative and KANK4-positive myofibroblasts may originate from distinct progenitor cells. Indeed, most myofibroblasts in keloids showed KANK4 expression; however, only a few myofibroblast in immature scars expressed KANK4. Notably, most myofibroblasts exhibited TAGLN expression. Additionally, the upregulation of KANK4 after TGF-β stimulation was markedly more significant in keloid fibroblasts versus immature scar fibroblasts (Fig. 4b). The scRNA-seq data showed that KANK4-positive fibroblasts are distinct from normal scar fibroblasts (Supplementary Fig. 3d,e). These data indicate that the origins of keloid myofibroblasts might differ from those of immature scars.

A limitation of this study is that, due to the absence of validation studies using animal models, we could not determine whether *KANK4* overexpression actually leads to the development of keloids in vivo. Further analysis is required to address this hypothesis. Nevertheless, our study provides a new perspective on the pathogenesis of keloids, and highlights KANK4 as a potential target for keloid.

### Supplementary Information

Supplementary Information 1.

Supplementary Information 2.

Supplementary Information 3.

Supplementary Information 4.

## Data Availability

Datasets related to this article have been submitted to an open-source data repository, Gene Expression Omnibus (GEO, https://www.ncbi.nlm.nih.gov/geo/, accession number GSE245660).

## Abbreviations

α-SMA: Alpha-smooth muscle actin
ECM: Extracellular matrix
FDR: False discovery rate
µm: Micrometer
scRNA-seq: Single-cell RNA sequencing
SD: Standard deviation of the mean
TAGLN: Transgelin
