# Supplementary material for: Exclusive expression of KANK4 promotes myofibroblast mobility in keloid tissues
Source: Sci Rep. 2024 Apr 16;14:8725. doi: 10.1038/s41598-024-59293-z (PMC11018845; doi:10.1038/s41598-024-59293-z)
Supplement: Supplementary file 2 — Supplementary Information 2. [file 41598_2024_59293_MOESM2_ESM.docx]

**SupplemENtary Figure Legends**

**Supplementary Figure 1**

Area of TAGLN-positive myofibroblasts (encircled by red lines) and whole scars (encircled by blue lines) for an immature scar (above) and a keloid (below). These areas were manually delineated using TissuemorphDP (Visiopharm, Hoersholm, Denmark). The thickness of scar tissue was measured perpendicularly from immediately below the epidermal basal layer to the adipose layer. The average of five random measurements was calculated for each sample (red, green, blue, yellow, and pink straight lines). Magnification: ×10. Scale bar: 2 mm.

TAGLN, transgelin.

**Supplementary Figure 2**

The TAGLN-positive areas utilized in the RNA-seq analysis. Immature scar samples (s1 and s2) and keloid samples (k1, k2, and k3) were stained with TAGLN. The TAGLN-positive regions (encircled with red dotted lines) were retrieved, and RNA was extracted for subsequent RNA-seq analysis. Scale bar: 500 μm.

RNA-seq, RNA sequencing; TAGLN, transgelin.

**Supplementary Figure 3**

**(a) Representative images of immunohistochemical analyses for OPCML in an immature scar and a keloid.** Magnification ×4 and ×20. Scale bar: 100 μm. **(b) Representative images of immunohistochemical analyses for S100A7 in an immature scar and a keloid.** Magnification ×4 and ×20. Scale bar: 100 μm. **(c) Representative images of immunohistochemical analyses for TAGLN and KANK4 in serial sections of keloids. Both the TAGLN-negative area and TAGLN-positive areas were magnified to display the staining pattern of KANK4.** Magnification ×10 and ×40. Scale bars: 100 μm. (d) UMAP of single-cell analysis data obtained from the GSE163973 dataset. (e) Single-gene UMAP plot of fibroblasts displaying the presence of TAGLN and KANK4.

KANK4, KN motif and ankyrin repeat domains 4; OPCML, opioid binding protein/cell adhesion molecule like; RNA-seq, RNA sequencing; S100A7, S100 calcium binding protein A7; TAGLN, transgelin; UMAP, uniform manifold approximation and projection.

**Supplementary Figure 4**

(a) mRNA expression levels of *ACTA2*, *TAGLN*, and *KANK4* in normal fibroblast (NFB1) after treatment with TGF-β (10ng/μl) for 24 h. Error bars show the mean ± SD. *P*-value was determined using the two-tailed *t*-test. * *P* < 0.05, ** *P* < 0.01. (b) Protein levels of EGFP-KANK4 after transfection of FB1, FB2 and FB3 with pcDNA3-EGFP (CTRL) or pcDNA3-EGFP-KANK4 (KANK4) for 48 h. The protein levels of β-actin were used as an internal control. (c) Cell proliferation assay for 5 days after transfection. The y-axis indicates relative absorbance at 450 nm wavelength on days 0-5. Error bars show the mean ± SD. (d) mRNA expression levels of *KANK4* after treatment with siKANK4 (100 nM) for 48 h. Error bars show the mean ± SD. *P*-value was determined using the two-tailed *t*-test. * *P* < 0.05. (e) Representative image of migrated cells in Transwell assay after transfection of cells with si negative control (siNC) or siKANK4 with 100 nM for 48 h. Scale bar: 250 μm (left). The bar graphs show the number of migrated cells, counted in each field under a microscope (right). Error bars show the mean ± SD. *P*-value was determined using the two-tailed *t*-test. *** *P* < 0.001. siNC, negative control siRNA .

**Supplementary Figure 5**

**(a)** mRNA expression levels of *COL1A2* and *COL3A1* after transfection with pcDNA3-EGFP (CTRL) or pcDNA3-EGFP-KANK4 (KANK4) for 48 h. Error bars show the mean ± SD. *P*-value was determined using the two-tailed *t*-test. (b) Protein levels of EGFP-KANK4 after transfection of immortalized endometrial fibroblasts SC10 with pcDNA3-EGFP (CTRL) or pcDNA3-EGFP-KANK4 (KANK4) for 48 h. The protein levels of β-actin were used as an internal control. (c) Representative images of migrated cells in Transwell assay after transfection with pcDNA3-EGFP (CTRL) or pcDNA3-EGFP-KANK4 (KANK4) for 48 h. Scale bar: 250 μm (left). The bar graphs show the number of migrated cells, counted in each field under a microscope (right). Error bars show the mean ± SD. *P*-value was determined by two-tailed *t*-test. **** *P*< 0.0001. (d) Cell proliferation assay for 5 days after transfection. The y-axis indicates relative absorbance at 450 nm wavelength on days 0-5. Error bars show the mean ± SD.

CTRL, control; EGFP, enhanced green fluorescent protein; GFP, green fluorescent protein; KANK4, KN motif and ankyrin repeat domains 4; SD, standard deviation.

**Supplementary Figure 6**

(a) KANK4 isoforms and Primer sets, as viewed in the UCSC Genome Browser. (b) The mRNA expression levels assessed using the KANK4 Primer set 2, were measured in the same samples shown in Figure 2d, immature scars (n = 10) and keloids (n = 12). Error bars show the mean ± SD. *P*-value was determined using the two-tailed *t*-test. ** *P* < 0.01. (c) Ratio of the expression levels of KANK4 Primer set 2 to Primer set 1. (d) Transwell assay after transfection with pcDNA3-EGFP (CTRL), EGFP-KANK4 (KANK4), or EGFP-KANK4-full length vector for 48 h. The bar graphs show the number of migrated cells, counted in each field under a microscope. Error bars show the mean ± SD. (e) Cell proliferation assay for 5 days after transfection. The y-axis indicates relative absorbance at 450 nm wavelength on days 0-5. Error bars show the mean ± SD.

CTRL, control; EGFP, enhanced green fluorescent protein; GAPDH, glyceraldehyde-3-phosphate dehydrogenase; KANK4, KN motif and ankyrin repeat domains 4; ns, not significant; SD, standard deviation; UCSC, University of California Santa Cruz.
