# Supplementary material for: Exclusive expression of KANK4 promotes myofibroblast mobility in keloid tissues
Source: Sci Rep. 2024 Apr 16;14:8725. doi: 10.1038/s41598-024-59293-z (PMC11018845; doi:10.1038/s41598-024-59293-z)
Supplement: Supplementary file 4 — Supplementary Information 4. [file 41598_2024_59293_MOESM4_ESM.docx]

**Table S1.** Clinical background of keloid patients and normal scar controls for gene expression analyses and fibroblast functional analyses.

| Sample ID | Scar type | Age | Sex | Race | Site of sampling | Duration after injury (years) | Assay type |
| --- | --- | --- | --- | --- | --- | --- | --- |
| s1 | immature scar | 60 | F | Asian | chest | 1.25 | RNA-seq |
| s2 | immature scar | 46 | F | Asian | chest | 0.75 | RNA-seq |
| s3 | immature scar | 73 | F | Asian | chest | 0.83 | qPCR |
| s4 | immature scar | 49 | F | Asian | chest | 0.5 | qPCR |
| s5 | immature scar | 41 | F | Asian | chest | 0.75 | qPCR |
| s6 | immature scar | 49 | F | Asian | chest | 1.25 | qPCR |
| s7 | immature scar | 55 | F | Asian | chest | 0.91 | qPCR |
| s8 | immature scar | 31 | F | Asian | chest | 0.58 | qPCR |
| s9 | immature scar | 27 | F | Asian | chest | 0.91 | qPCR |
| s10 | immature scar | 49 | F | Asian | abdomen | 1.5 | qPCR |
| s11 | immature scar | 38 | F | Asian | chest | 0.83 | qPCR |
| s12 | immature scar | 47 | F | Asian | chest | 0.75 | qPCR |
| k1 | keloid | 28 | F | Asian | ear | 2 | RNA-seq |
| k2 | keloid | 64 | M | Asian | abdomen | 3 | RNA-seq |
| k3 | keloid | 74 | M | Asian | shoulder | 10 | RNA-seq |
| k4 | keloid | 53 | M | Asian | ear | 10 | qPCR |
| k5 | keloid | 29 | F | Asian | ear | 4 | qPCR |
| k6 | keloid | 67 | F | Asian | chest | 3 | qPCR |
| k7 | keloid | 47 | F | Asian | shoulder | 3 | qPCR |
| k8 | keloid | 26 | F | Asian | ear | 10 | qPCR |
| k9 | keloid | 27 | F | Asian | ear | 3 | qPCR |
| k10 | keloid | 52 | F | Asian | abdomen | 5 | qPCR |
| k11 | keloid | 88 | M | Asian | abdomen | 4 | qPCR |
| k12 | keloid | 40 | F | Asian | abdomen | 3 | qPCR |
| k13 | keloid | 66 | F | Asian | abdomen | 16 | qPCR |
| k14 | keloid | 21 | F | Asian | ear | 1.5 | qPCR |
| k15 | keloid | 36 | F | Asian | neck | 3 | qPCR |
| NFB1 | normal skin | 42 | F | Asian | abdomen | - | cell culture |
| FB1 | immature scar | 28 | M | Asian | chest | 1 | cell culture |
| FB2 | keloid | 31 | F | Asian | ear | 9 | cell culture |
| FB3 | keloid | 30 | M | Asian | ear | 1 | cell culture |

**Table S2.** Primer sequences for qPCR.

| Target gene | Primer sequence (5' to 3') |
| --- | --- |
| OPCML | Forward: GAGTCCTGGGAAGTTGTGGC |
|  | Reverse: GTCATCTATGGTACACCTGAGGG |
| S100A7 | Forward: CCAAGCCTGCTGACGATGA |
|  | Reverse: GACATCGGCGAGGTAATTTGTG |
| S100A8 | Forward: GTCTTTCAGAAGACCTGGTGGG |
|  | Reverse: GAGGACACTCGGTCTCTAGC |
| KRT6A | Forward: AGAGCTCAGAGGCATGCAG |
|  | Reverse: GCAGCATCCACATCCTTCTTC |
| KANK4 | Forward: GCAGCTCGCCAAGAACCTT |
|  | Reverse: CTCCATCTTTGGAGCGCTGA |
| KANK4- | Forward: GTGCAGGAGGTAATGGGACC |
| Primer2 | Reverse: ACACTTCTTCTCTGCCTCGC |
| PTPRD | Forward: CTGCCTGTGGATCAAGTGTT |
|  | Reverse: TAACTTCTGTGTGGCTCGGC |
| ELN | Forward: AAGCCGCCCAGTTTGGGTTA |
|  | Reverse: CAACTCCACCAGGGCCAATG |
| TNXB | Forward: ACCTTCACCACAGGGCTAGA |
|  | Reverse: CTCAGTCCAAGTGAGCAGGG |
| TAGLN | Forward: CATCCTGTCTGTCCGAACCC |
|  | Reverse: GACTGAGAGGGTGGGTTTCC |
| ACTA2 | Forward: TATCCCCGGGACTAAGACGG |
|  | Reverse: CACCATCACCCCCTGATGTC |
| COL1A2 | Forward: GGTGGTGGTTATGACTTTGG |
|  | Reverse: GTTCTTGGCTGGGATGTTTT |

**Table S3.** Differentially expressed genes from RNA-sequence analysis (GSE113619) and microarray analysis (GSE92566).

|  |  | **Keloids vs Immature scars (GSE113619)** | |  | **Keloids vs Normal skin (GSE92566)** | |
| --- | --- | --- | --- | --- | --- | --- |
| **Gene name** | **Ensembl ID** | **log_2_FC** | **p-value** |  | **log_2_FC** | **p-value** |
| CDR1 | ENSG00000184258 | 6.733203 | 0.005306 |  | 1.01491 | 0.025964 |
| S100A7A | ENSG00000184330 | 2.85686 | 9.79E-07 |  | 5.211461 | 0.042694 |
| S100A9 | ENSG00000163220 | 2.109368 | 3.87E-07 |  | 3.857051 | 0.045136 |
| MMP1 | ENSG00000196611 | 2.032122 | 2.40E-08 |  | 2.347231 | 0.025157 |
| S100A7 | ENSG00000143556 | 1.848036 | 6.20E-06 |  | 1.47365 | 0.039341 |
| GRP | ENSG00000134443 | 1.659762 | 0.004669 |  | 3.770232 | 0.003295 |
| OPCML | ENSG00000183715 | 1.646878 | 8.26E-10 |  | 5.608848 | 0.001384 |
| SLC24A2 | ENSG00000155886 | 1.27439 | 1.95E-07 |  | 2.539824 | 0.006408 |
| AKR1B10 | ENSG00000198074 | 1.270124 | 1.09E-08 |  | 3.206402 | 0.043818 |
| BMPR1B | ENSG00000138696 | 1.134461 | 1.51E-06 |  | 1.701686 | 0.011025 |
| RUNX2 | ENSG00000124813 | 1.107982 | 8.57E-07 |  | 2.376133 | 0.002671 |
| LYPD1 | ENSG00000150551 | 1.091887 | 1.18E-05 |  | 1.891661 | 0.047892 |
| COL11A1 | ENSG00000060718 | 1.080505 | 2.06E-05 |  | 5.359858 | 0.009089 |
| SALL1 | ENSG00000103449 | 1.071903 | 0.027144 |  | 4.832568 | 0.000428 |
| KANK4 | ENSG00000132854 | 1.047065 | 6.92E-05 |  | 3.028122 | 0.006097 |
| PTPRD | ENSG00000153707 | 1.030291 | 7.02E-06 |  | 2.156123 | 0.006514 |
| EDIL3 | ENSG00000164176 | 1.010836 | 3.86E-06 |  | 1.634971 | 0.002592 |
| TNFSF4 | ENSG00000117586 | 1.008836 | 1.96E-05 |  | 4.321946 | 0.00123 |
| ZBTB16 | ENSG00000109906 | -1.03714 | 0.000204 |  | -4.73797 | 0.000395 |
| NHLH1 | ENSG00000171786 | -1.0571 | 0.006265 |  | -1.46212 | 0.015351 |
| DKK1 | ENSG00000107984 | -1.09773 | 2.91E-05 |  | -3.64204 | 0.001246 |
| LMO3 | ENSG00000048540 | -1.10291 | 9.93E-06 |  | -4.56013 | 0.00062 |
| TOX3 | ENSG00000103460 | -1.17749 | 0.005312 |  | -1.48361 | 0.011557 |
| MATN4 | ENSG00000124159 | -1.22202 | 7.08E-05 |  | -1.71344 | 0.018252 |
| CLDN11 | ENSG00000013297 | -1.26769 | 1.17E-13 |  | -1.11643 | 0.010463 |
| GSTM5 | ENSG00000134201 | -1.33488 | 2.35E-06 |  | -1.38868 | 0.022797 |
| CLCNKB | ENSG00000184908 | -1.65713 | 0.032222 |  | -3.12321 | 0.004738 |
| PCSK1N | ENSG00000102109 | -2.60582 | 4.95E-07 |  | -2.85819 | 0.020064 |
| DLX2 | ENSG00000115844 | -3.14531 | 0.009292 |  | -2.48974 | 0.010488 |
